# Identification of unannotated exons of low abundance transcripts in *Drosophila melanogaster *and cloning of a new serine protease gene upregulated upon injury

**DOI:** 10.1186/1471-2164-8-249

**Published:** 2007-07-24

**Authors:** Rafaela M Maia, Valeria Valente, Marco AV Cunha, Josane F Sousa, Daniela D Araujo, Wilson A Silva, Marco A Zago, Emmanuel Dias-Neto, Sandro J Souza, Andrew JG Simpson, Nadia Monesi, Ricardo GP Ramos, Enilza M Espreafico, Maria L Paçó-Larson

**Affiliations:** 1 Departamento de Biologia Celular, Molecular e de Bioagentes Patogênicos, Faculdade de Medicina de Ribeirão Preto, Universidade de São Paulo, Ribeirão Preto 14049-900, SP, Brazil; 2 Departamento de Genética e Centro de Terapia Celular, Faculdade de Medicina de Ribeirão Preto, Universidade de São Paulo, Ribeirão Preto 3900 14049-900, SP, Brazil; 3 Departamento de Clínica Médica e Centro de Terapia Celular, Faculdade de Medicina de Ribeirão Preto, Universidade de São Paulo, Ribeirão Preto 3900 14049-900, SP, Brazil; 4 Ludwig Institute for Cancer Research, Rua Professor Antonio Prudente, 109, São Paulo 01509-010, SP, Brazil; 5 DDA: Faculdade de Medicina, Universidade de Ribeirão Preto-UNAERP, Av. Costabile Romano 14096-900 Ribeirão Preto, SP, Brazil; 6 EDN: Laboratório de Neurociências (LIM-27) Instituto de Psiquiatria, HCFMUSP, R. Ovidio Pires de Campos, s/n 05403-010, São Paulo, SP, Brazil and University of Texas/MD Anderson Cancer Center, 1515 Holcombe Blvd, 77030, Houston, TX, USA; 7 AJGS: Ludwig Institute for Cancer Research 605 Third Avenue New York, NY 10158, USA; 8 Departamento de Análises Clínicas, Toxicológicas e Bromatológicas, Faculdade de Ciências Farmacêuticas de Ribeirão Preto, Universidade de São Paulo, 14040-903 Ribeirão Preto, SP, Brazil

## Abstract

**Background:**

The sequencing of the *D.melanogaster *genome revealed an unexpected small number of genes (~ 14,000) indicating that mechanisms acting on generation of transcript diversity must have played a major role in the evolution of complex metazoans. Among the most extensively used mechanisms that accounts for this diversity is alternative splicing. It is estimated that over 40% of *Drosophila *protein-coding genes contain one or more alternative exons. A recent transcription map of the *Drosophila *embryogenesis indicates that 30% of the transcribed regions are unannotated, and that 1/3 of this is estimated as missed or alternative exons of previously characterized protein-coding genes. Therefore, the identification of the variety of expressed transcripts depends on experimental data for its final validation and is continuously being performed using different approaches. We applied the Open Reading Frame Expressed Sequence Tags (ORESTES) methodology, which is capable of generating cDNA data from the central portion of rare transcripts, in order to investigate the presence of hitherto unnanotated regions of *Drosophila *transcriptome.

**Results:**

Bioinformatic analysis of 1,303 *Drosophila *ORESTES clusters identified 68 sequences derived from unannotated regions in the current *Drosophila *genome version (4.3). Of these, a set of 38 was analysed by polyA^+ ^northern blot hybridization, validating 17 (50%) new exons of low abundance transcripts. For one of these ESTs, we obtained the cDNA encompassing the complete coding sequence of a new serine protease, named SP212. The *SP212 *gene is part of a serine protease gene cluster located in the chromosome region 88A12-B1. This cluster includes the predicted genes CG9631, CG9649 and CG31326, which were previously identified as up-regulated after immune challenges in genomic-scale microarray analysis. In agreement with the proposal that this *locus *is co-regulated in response to microorganisms infection, we show here that SP212 is also up-regulated upon injury.

**Conclusion:**

Using the ORESTES methodology we identified 17 novel exons from low abundance *Drosophila *transcripts, and through a PCR approach the complete CDS of one of these transcripts was defined. Our results show that the computational identification and manual inspection are not sufficient to annotate a genome in the absence of experimentally derived data.

## Background

Genome sequence determination of the model organism *Drosophila melanogaster *was a landmark that launched a new era for functional genomic studies in complex organisms. The almost complete version of the euchromatic DNA sequence was first released in March 2000 due to a collaborative effort of the *Drosophila *Genome Projects and Celera Genomics [1]. Using gene prediction softwares in combination with searches of protein and EST databases, initial *in silico *analyses indicated the existence of 13,601 protein-coding genes (PCG), an extraordinarily small number of genes when compared to the approximately 19.000 PCG encoded in the *C.elegans *genome [1].

After the release 1, an intensive collective work took place in order to improve sequence quality and annotation, fill in the gaps, and correct the assembly. With the aim of generating the information necessary to define the transcripts encoded in the genome, the Berkeley *Drosophila *Genome Project (BDGP) initiated a high throughput production of both EST and full length cDNA sequences based on conventional and normalized cDNA libraries from different tissues and developmental stages [2]. This effort was followed by non-BDGP projects with a major contribution from the Exelixis *Drosophila melanogaster *EST project, which has adopted sequencing of random primed libraries of mixed stage embryos, imaginal disks, and adult heads to increase the transcription units coverage [3]. Currently, there are about 39,346 full length mRNA and 532,557 EST sequences available in the NCBI database, totalizing approximately 16,681 clusters according to UniGene [4]. Since the year 2000, several subsequent genome versions have been released, each one improved by BDGP and annotated by FlyBase [5]. Release 3.2, considered the first finished version, was published in March 2004 and provided a complete revision of all gene models and other genome features [6], estimating a total number of 13,792 PCGs plus 527 non-protein coding genes (tRNAs, rRNAs, microRNAs, sn/snoRNAs). Release 4.3, the last annotated genome version published in March 2006, includes a total of 14,816 genes and is available for searches by gene annotation, BLAST or sequence ID at the FlyBase website [5].

During the last few years, an enthusiastic debate about the number of PCGs in the organisms with sequenced genomes has arisen. For *D. melanogaster*, estimates varied from the initial ~ 13,600 coding gene predictions [7] to about 16,000 gene predictions, based on microarray expression data [8]. A careful computational and experimental analysis carried to validate the *Drosophila *genome annotation has recently concluded that the *D.melanogaster *genome in fact contains approximately 14,000 protein-coding genes, although some genes presenting unusual features that make them refractory to prediction methods may remain to be discovered [9]. However, the truthful notion about the complexity of the *D. melanogaster *transcriptome is still under construction. In this respect, it has been inferred from DNA oligonucleotide microarrays, with unique sequences tiled throughout the genome and across predicted splice junctions, that over 40% of the *Drosophila *genes contain one or more alternative exons [10]. Additionally, a transcription map with a 35 bp resolution of the initial 24 hours of development indicates that 30% of the transcribed regions are still unannotated. Approximately 23% of these are intronic and 7% correspond to intergenic regions. Based on manual and computational surveys designed to identify coregulated expression patterns between unannotated and annotated genome regions, it was estimated that 29% of the unannotated regions are part of transcripts incompletely annotated or potential alternative exons of known genes [11]. Therefore, correcting and refining the genome annotation is a reiterative task, which is continuously being done and depends on experimental data for final validation, especially for the identification of rare transcripts and alternative splice variants. With the aim of covering the diversity of transcripts expressed in *Drosophila *the generation of EST information from different sources is currently under way [2,3].

Here we use the Open Reading Frame Expressed Sequence Tags (ORESTES) methodology, which is based on low stringency RT-PCR, to generate *D. melanogaster *expressed sequence information. ORESTES are preferentially derived from the central coding portions of the transcript and frequently identify less abundant messages [12,13]. Such approach was previously applied for human transcriptome characterization, validating a large percentage of genes and identifying 219 unannotated transcribed sequences on chromosome 22 [14]. More recently, a large-scale analysis of ORESTES derived from head, neck and thyroid tumors pointed to 788 putative new alternative splicing isoforms. A subset of 34 was submitted to experimental validation resulting in the confirmation of 23 (68%) new alternate exons [15].

Analysis of 1,303 *Drosophila *ORESTES clusters revealed 68 potential transcribed regions unannotated in the current version of the genome (release 4.3). Experimental validation of 38 (~ 50%) of this unannotated ORESTES revealed 17 new exons that most likely belong to low abundance transcripts. Using the ORESTES information together with a PCR based approach we obtained the complete coding sequence of a new serine protease which mRNA expression is induced upon infection. Our data reinforce the importance of PCR based methodologies for refining the *Drosophila *transcriptome, particularly for the identification of previously unannotated low copy transcripts.

## Results and discussion

### ORESTES *in silico *analysis

**Figure 1:**
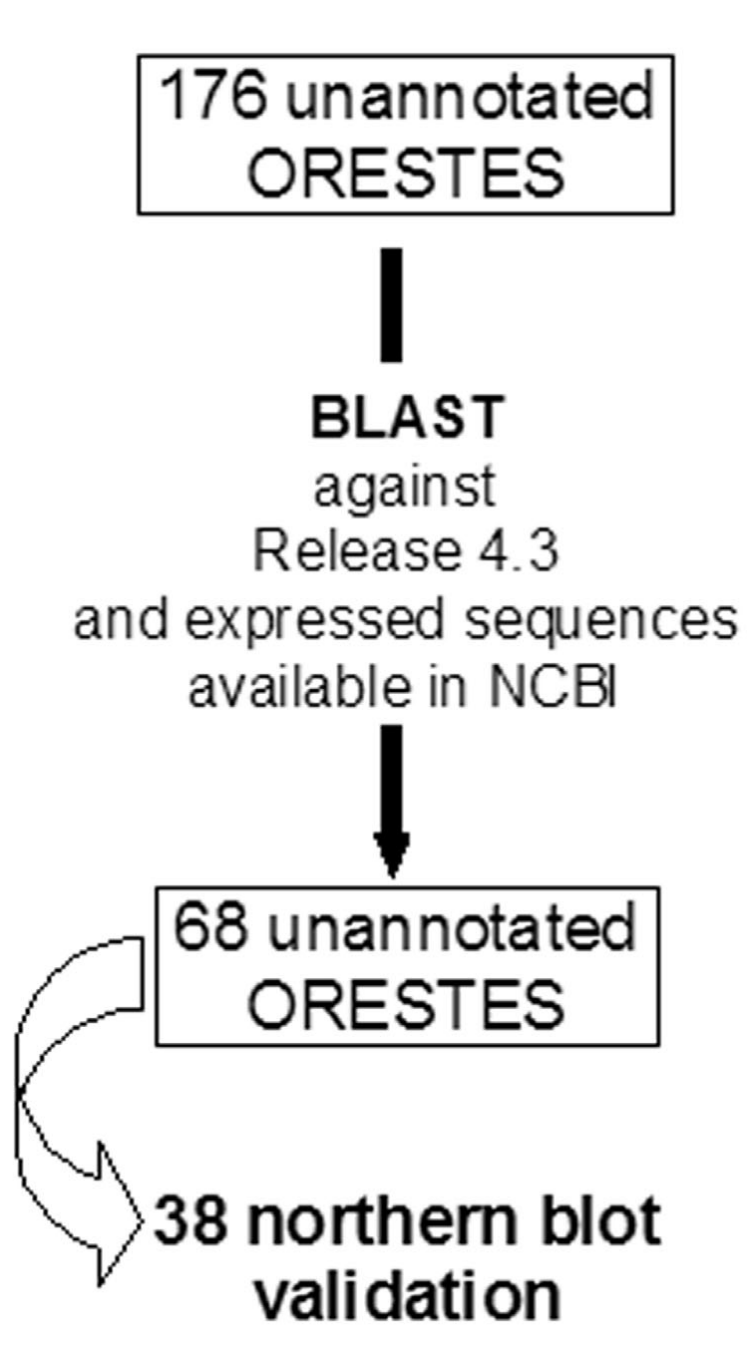
**Analysis flowchart of 176 ORESTES unannotated in *Drosophila melanogaster *genome version 1**. Analysis against the genome assembly 4.3 and expressed sequences were manually performed using BLASTn at the FlyBase [5] and BLAT Search [16].

Of 1,303 *Drosophila *ORESTES clusters from different developmental stages of *D.melanogaster*, 176 were identified as unannotated in the genome release 1 (see material and methods). We re-analysed this set of 176 ORESTES against the current annotated version of the genome (Figure 1). Sixty-eight of these clusters aligned in unannotated genome regions indicating putative new exons (GeneBank_Accn. EG974084 to EG974151). Fifty-three out of the 68 clusters either match or overlap ESTs generated by other projects [2,3], suggesting that they indeed represent true transcripts. Amongst the remaining clusters, 98 overlap annotated exons represented by cloned cDNAs and ESTs, 8 correspond to transposons or repetitive sequences and 2 are chimeric. About half of the 68 unannotated ORESTES (38) were selected for validation by developmental northern blot hybridization.

### 50% of the unannotated exons detected by ORESTES belong to low abundance transcripts

**Figure 2:**
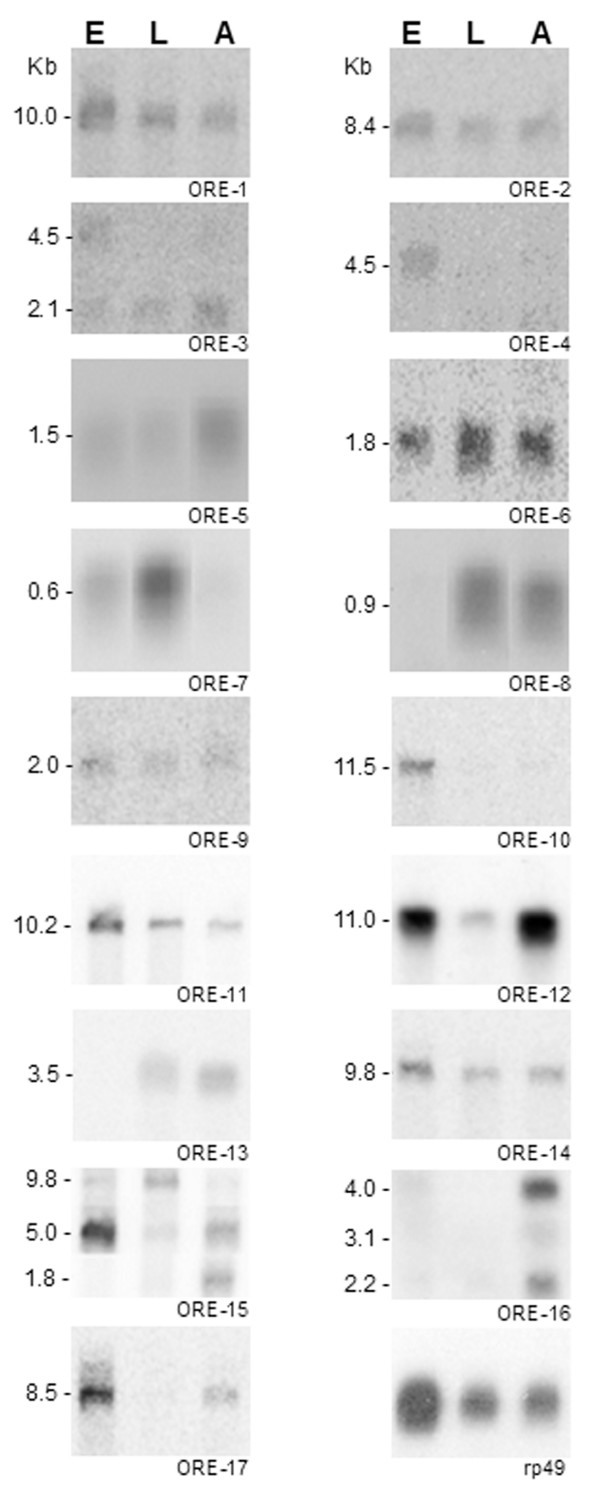
**Developmental profile of transcripts containing exons unannotated in the current version (4.3) of the *D.melanogaster *genome**. Autoradiograms of Northern blots containing poly A+ RNA extracted from embryos (E), third instar larvae (L3), and adults (A), which were hybridized to the probes indicated at the bottom of each blot. The estimated size (kb) of each transcript is indicated on the left.

Of the 38 ORESTES that mapped in unannotated exons of the version 4.3 of the genome, 17 (50%) detected mRNAs only in polyA^+ ^northern blots (Figure 2) indicating that they all represent low abundance mRNAs. The other 21 ESTs could also represent transcribed regions that cannot be detected by our analysis, such as precursors of small non-coding RNAs [17]. Among the 17 validated ORESTES, 4 are derived from the embryo library (DE), 3 are from the library constructed with RNA extracted from larvae, prepupae plus pupae (DL), 5 are from an adult library (DA) and 5 are from a library constructed with a mixture of the RNA from all developmental stages (DP). Based on these data and in the number of ORESTES clusters produced from the DE (113), DL (430), DA (887) and DP (729) libraries, the estimated discovery rate of new exons for each library was 35.4, 7.0, 5.6 and 6.9 new exons per 1,000 ORESTES from the DE, DL, DA and DP libraries, respectively. The 5–6 higher discovery rate found for DE library indicates that, despite the large amount of embryo ESTs available in the public database, the embryonic transcriptome needs further sequencing for its definition, which is in accordance with previous transcriptional analysis of the *Drosophila *embryogenesis performed at the genomic scale [11].

The majority of the validated ORESTES (14) detected a single band in northen blots, which could indicate the presence of unique transcripts. Three ORESTES detected more than one mRNA species (Figure 2, ORE-3, ORE-15, ORE-16), which could either constitute isoforms of the same gene or mRNAs encoded by different genes sharing common exons. Ten of these 17 validated ORESTES detected transcripts in all analysed developmental stages, namely: embryo, larvae and adult (Figure 2; ORE-1, -2, -3, -5, -6, -9, -11, -12, -14, -15). The other seven (Figure 2; ORE -4, -7, -8, -10, -13, -16, -17) detected stage specific transcripts. ORE-4 and ORE-10 detect mRNAs of 4.5 and 11.5 kb, respectively, which are mainly expressed in embryos. The 8.5 kb mRNA detected by ORE-17 is abundant in embryos and present in lower amounts in adults. ORE-12 detects an 11.0 kb transcript present mainly in embryo and adults. The transcripts of about 0.9 kb and 3.5 kb detected by ORE-8 and ORE-13, respectively, were only detected in larvae and adults. ORE-7 detects a 0.6 kb mRNA present at higher levels in larvae that is also expressed in embryos. ORE-3 detected one mRNA of about 4.5 kb exclusively expressed in embryos, and a 2.1 kb mRNA present at all stages. ORE-15 detected three mRNAs of 9.8, 5.0 and 1.8 kb. The 9.8 kb mRNA is mainly detected in larvae; the 5.0 kb RNA is much more abundant in embryos, but also detected in adults; and the 1.8 kb RNA is exclusively expressed in adults. ORE-16 detected 3 different transcripts of about 4.0, 3.1 and 2.2 kb, all of them observed in adult flies.

Altogether, our ORESTES analyses resulted in the validation of 17 novel exons. Of these, five ESTs constitute, up to now, the unique expressed sequence information for the corresponding genomic regions (Table 1), despite the wealth of cDNA data available for *Drosophila*, and the ongoing projects of cDNA/EST generation using tissue specific and normalized cDNA libraries [3,18,19]. Both strands of the longest EST representing each one of the 17 validated ORESTES clusters were sequenced. Alignment of these complete sequences with the *Drosophila *genome show that 8 of them map to intergenic regions, 6 map to introns of annotated genes, two align in intron/exon boundaries and another partially overlaps the last exon of an annotated gene (Table 1). With the exception of ORE-13, that lies more than 14 kb from the closest annotated gene, the other ORESTES mapping in intergenic regions (ORE-2, -3, -4, -10, -11, -12, -17) are located less than 1 kb of the last exon of the nearest gene, and could represent a novel alternate 3'UTR of these genes. If this were the case, the novel isoforms would be larger than the annotated transcripts since their sizes are much shorter than the detected mRNAs. ORE-16 overlaps the last exon of the CG13165, whose CDS is incomplete, and therefore could represent an extension of this gene. Additionally, ORE-16 detected three transcripts longer than the CG13165 predicted transcript, indicating that this gene might express different mRNA isoforms. Of the five ORESTES located in introns, four (ORE-7, -8, -9, -15) detected mRNAs shorter than the transcripts mapped in the same region and could represent exons of other isoforms. ORE-5 and ORE-6 map to an exon-intron boundary of electronically predicted genes and could either represent a different isoform or the correct structure of the transcription units. However, since ORESTES are not strand-specific, they can also represent exons of unannotated genes encoded in the opposite strand of the same genomic region. Interestingly, all the new annotated exons belong to low abundance messages confirming the ability of ORESTES in identifying rare new transcripts [13].

**Table 1:** Genomic mapping of the validated ORESTES and sizes of the respective transcripts

**ORE**	**GeneBank_Accn**	**Cytogenetic map**	**Exon/Intron/Intergenic**	**Transcript**^α^**sizes **(kb)	**Closest annotated gene**	**Predicted transcript sizes^β ^of the closest gene**
1	EG974100	7D3–5	Intron	10.0	*fs(1)h*	4.0
2	EG974085	88D1	Intergenic	8.4	CG33967	4.9
3	EG974101	25A3	Intergenic	2.1; 4.5	CG11928	0.3
4	EG974134^•^	32A2	Intergenic	4.5	CG7329	1.6
5	EG974091^•^	88A12	Exon/Intron	1.5	CG33329	1.2
6	EG974139	66A22-B3	Exon/Intron	1.8	nmo (CG7892)	Isoforms 2.2–3.1
7	EG974151	87E8	Intron	0.6	CG9813	Isoforms 2.1–2.8
8	EG974118	91C6-D1	Intron	0.9	CG7720	Isoforms 2.4–2.5
9	EG974120	9F2–4	Intron	2.0	Imp (CG1691)	Isoforms 2.8–3.9
10	EG974125	33A1–2	Intergenic	11.5	CG18265	6.4
11	EG974117	98F10	Intergenic	10.2	CG11874	3.2
12	EG974128	74E4–5	Intergenic	11.0	TORC	3.0
13	EG974088^•^	25D6-E1	Intergenic	3.5	nompC (CG11020-RB)	5.1
14	EG974149	74D4	Intron	9.8	CycT (CG6292)	Isoforms 3.2–4.3
15	EG974105^•^	33A1–2	Intron	1.8; 5.0; 9.8	crol (CG14938)	Isoforms 6.2–7.1
16	EG974142^•^	48F6	Exon/Intergenic	2.2; 3.1; 4.0	CG13165	1.5
17	EG974111	90D1-E1	Intergenic	8.5	cpo (CG31243)	Isoforms 2.8–6.2

^α ^sizes of the transcripts detected by ORESTES; ^β^sizes of the predicted transcripts encoded by genes located closest to the ORESTES mapping site, ^• ^do not match any EST/cDNA sequence available up to October 2006 in the NCBI database

### Cloning a new *D. melanogaster *serine-protease – SP212

**Figure 3:**
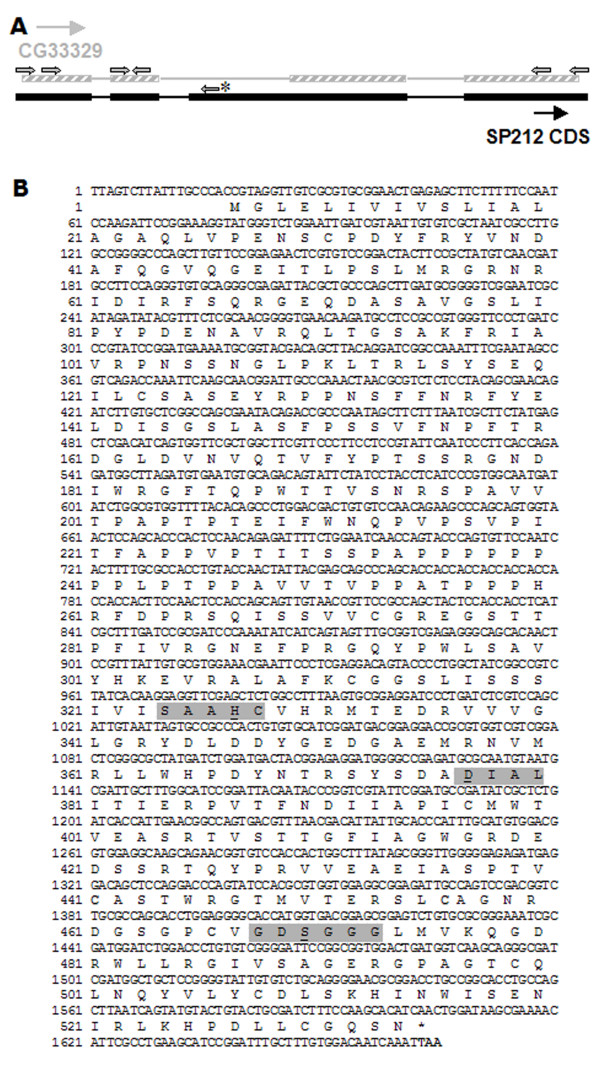
**Molecular characterization of a new serine endopeptidase gene**. A) Graphic representation of the genomic alignment of the new transcript (SP212) identified by ORE-5. The small arrows localize the primers used for cloning the SP212 cDNA. The asterisk indicates the primer that was based on the ORE-5 sequence. CG-33329 was electronically annotated. B) Sequence of the SP212 cDNA and the amino-acid sequence encoded in the largest open reading frame (GenBank accession number: EF108315). Grey boxes mark the *motifs *containing the SP catalytic residues, which are underlined.

Although representing partial transcripts, ORESTES information can be useful in the process of transcriptome finishing [20]. Here we illustrate this potential by cloning a cDNA, which we could not isolate after several attempts of screening cDNA libraries. The chosen transcript was first detected by ORE-5 that maps in an intron/exon boundary of a computationally predicted gene (CG33329). Based on the sequence of ORE-5, together with the exons and the genomic sequences flanking this genomic region, we designed primers with the aim of amplifying fragments covering at least the full coding sequence (CDS) of the gene (Figure 3A). We have generated 3 overlapping fragments, derived from the same transcript, that were completely sequenced producing a consensus sequence of 1,664 nt (figure 3B). This transcript presents 4 exons, as for the CG33329 predicted transcript, but the third exon is longer in the cloned cDNA (Figure 3). Despite several attempts using mRNA from animals at all developmental stages we could not confirm the existence of the predicted CG33329 and we suggest that the cloned sequence probably represents the correct transcription unit of CG33329. This consensus has an open reading frame of 1.584 nt encoding a 528 amino acids polypeptide (figure 3B). The deduced protein has an estimated molecular weight of 57.8 kDa and an isoeletric point of 6.41. Using BLASTx tool we found that this polypeptide presents significant identity with serine proteases (SP), thus revealing a new *D. melanogaster *SP protein.

SP proteins are proteolytic enzymes that require serine for their catalytic activity. They are ubiquitous peptidases, which perform a wide array of important physiological functions, including digestion, blood coagulation, fibrinolysis, cellular and humoral immunity, fertilization and embryonic development [21]. In a previous work that intended to map all SPs and SP related proteins in the genome of *D. melanogaster*, Ross and colleagues [22] performed a series of similarity searches (PSI-BLAST) and found a total of 211 GenBank entries encoding SPs and SPHs (SP homologs – proteins in which one or more residues of the catalytic triad are missing). Following the nomenclature criteria used by these authors, we named the additional SP gene characterized here as SP212. PROSITE analysis [23] revealed that *D. melanogaster *SP212 presents the conserved catalytic triad ordered His, Asp, Ser (HDS), a characteristic of SP chymotrypsin family. These residues form two diads, Ser-His and His-Asp, that operate in concert for the acyl mechanism of catalysis [24]. These catalytic residues are, as in most SPs, embedded into highly conserved motifs: SAAHC, DIAL and GDSGGG. SP212 also presents the signal peptide sequence in its amino terminal end with the cleavage between residues 17 and 18, an indication that this peptidase might be secreted (figure 3B).

**Figure 4:**
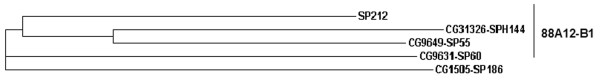
**SP212 was probably originated through gene duplication during evolution of SP family**. The phylogram is based on multiple alignment of the amino acid sequences of the catalytic domain of SP212 and other 3 SPs and 1 SPH of *Drosophila*. These sequences are from proteins that presented the highest similarity with SP212 catalytic domain sequence (e-value < -20) in searches against the NCBI databank, using Blastx. The alignment was performed using Clustalw (20). SP55, SPH144 and SP60, as well SP212, localize in the same region (88A12-B1) of chromosome 2R, whereas SP186 is located in chromosome X.

The SP212 gene is located in a chromosomal region (88A12-B1) that contains three predicted genes, CG9631, CG9649 and CG31326, which codify two SPs and one SPH respectively, named: SP60, SP55 and SPH144. In order to evaluate the similarity level between SP212 and related proteins, we performed a multiple alignment comparison with a selected group of SPs and SPHs. The protein sequences for this alignment were selected based on their similarity (E value < -20) with SP212 using the BLASTx tool. Multiple alignment [25] of the catalytic domain of these proteins (50 residues upstream of the conserved His and 50 residues downstream of the Ser catalytic site) showed that SP212 is more closely related to SP55 and SPH144, which are located in the 88A12-B1 chromossomic region. Thus, SP212, SP55 and SPH144 genes were possibly generated through gene duplication during evolution of the SP family, probably being derived from a common ancestor (figure 4).

### SP212 gene is upregulated in response to infection

**Figure 5:**
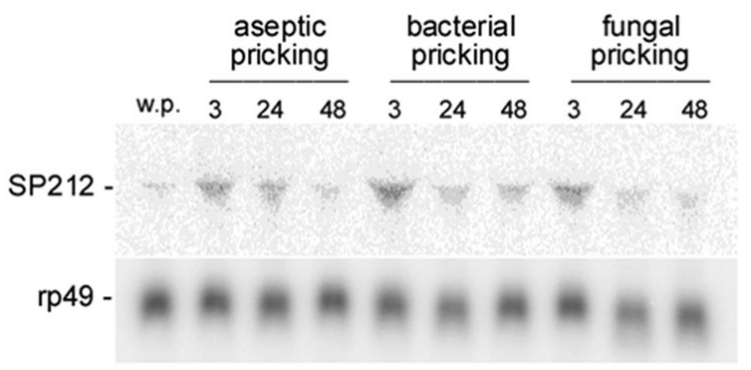
**Induction of the SP212 mRNA in adult flies after infection with different microorganisms**. Northern blot analysis of total RNA extracted from adult flies at different times (3, 24 and 48 hours) after challenging by pricking with a needle dipped into 10^9 ^cells/ml cultures of either Gram^+ ^(*S.aureus*) or Gram^- ^(*E.coli*) bacteria or fungi (*A.fumigattus*). Note that asceptic pricking by itself triggers the induction of the SP212, albeit at a lower level.

SP212 is 528 amino acid residues long and presents the potential to form three disulfide-bridges. These structural features of SP212 indicate that this enzyme is probably associated with physiological functions other than digestion. Digestive SPs are much smaller, contain approximately 250 amino acid residues and have a relatively simpler structure, with a short amino-terminal activation peptide connected to a catalytic domain. Additional domains in larger enzymes allow protein-protein interactions, which are usually needed for regulating their activity and specific localization. The presence of a disulfide-bridge structure, called the clip domain, for example, is characteristic of arthropod SPs and SPHs that are involved in defense response and embryonic development [26]. Interestingly, data of high-density oligonucleotide microarrays assaying nearly every *Drosophila *gene indicated that the SP55, SP60 and SPH144 encoding genes, mapped at the *locus *88A12-B1 (CG9649, CG31326 and CG9631), are all upregulated upon septic injury with a mixture of Gram-negative (*E.coli*) and Gram-positive (*M.luteus*) bacteria, or to natural infection with the entomopathogenic fungus *B.bassiana *[27]. Additionally, there are evidences that CG9631 (SP60) is controlled by Toll and CG31326 (SPH144) by both Toll and Imd signalling pathways [28], the major regulators of immune response in *D. melanogaster*. These observations lead to the suggestion that SP212, which is localized in the same genomic cluster, could also be involved in defense responses. To test this hypothesis we analysed the effect of aseptic injury and septic injuries with gram-positive (*S. aureus*) and gram-negative (*E. coli*) bacteria and fungi (*A.fumigatus*) on the SP212 levels of expression at different time points after the injuries. As shown in figure 5, we observed that the amounts of SP212 mRNA increase upon infection, with the highest levels being detected 3 hours after injuries with either bacteria or fungi. The increase of SP212 mRNA amounts is also observed after aseptic pricking, albeit in lower amounts than in the animals challenged with microorganisms, similarly to what has been previously described for antimicrobial peptide genes [29]. Therefore, the SP212 gene and the genes encoding the other three serine proteases mapped in the *locus *88A12-B1 seem to be co-regulated in response to injury and probably have a role in *D. melanosgaster *defense against pathogens. Recent genetic [30,31] and *in vivo *RNAi studies [32-34] have provided information about the role of ten *Drosophila *SP/SPH in septic injury. To our knowledge no further functional studies about the SP/SPH clustered in the 88A12-B1 chromosome region have been reported so far. It will be important to characterize the injury-response elements involved in this *locus *activation as well as to determine the function of these new enzymes in the defense response.

## Conclusion

The analysis of a relatively small set of ORESTES allowed the validation of 17 novel exons present in low abundance transcripts, and led to the cloning of a new serine peptidase that is induced during the defense response. These results illustrate the importance of PCR-based approaches as complementary tools for the identification of transcribed regions in sequenced genomes. The final determination of any transcriptome is not a trivial task and one might envisage the occurrence of rare transcripts that will be missed by conventional cloning.

## Methods

### Biological samples, ORESTES preparation and sequencing

Dechorionated embryos, larvae plus prepupae and pupae as well as adult flies were collected from an isogenic *y, w*^1118 ^stock of *Drosophila melanogaster*, immediately frozen in liquid nitrogen and stored at -80 C until use. Total RNA was extracted with TRIZOL and poly(A)^+ ^RNA was isolated (MiniMacs; Miltenyi Biotec). The RNA quality was assessed by northern blot hybridization using a *Drosophila *α1-tubulin probe [35]. High quality total RNA preparations were further treated with DNase I (Promega). The absence of DNA contaminants was assessed by Southern blot hybridization of PCR amplification products using *Drosophila *mitochondrial DNA primers. Template preparations were performed as described by Dias-Neto and colleagues [12] with some minor modifications as follows: 15 ng of purified mRNAs were used for cDNA synthesis and amplification, using RT-PCR beads (Amershan-Pharmacia Biotech, USA) and a set of randomly selected oligonucleotide primers (15 to 20 mers). ORESTES profiles were generated after a cDNA synthesis step at 42°C for 60 mins immediately followed by cDNA denaturation at 75°C and amplification by PCR using a multiple annealing step. The annealing was performed for 10 secs at each temperature and the temperatures varied from 66°C to 44°C (with progressive reductions of 2°C), within each cycle. Primer extension was performed at 72°C for 1 min and denaturing at 95°C for 45 secs in 40 cycles. A final extension step at 72°C for 7 mins was undertaken. Profiles composed of a DNA smear were size selected in order to separate amplification products of distinct size ranges, varying from 0.3 to 1.5 kb. The fragments were ligated into pUC18 using Sureclone (Amersham-Pharmacia) and the recombinant plasmids used for bacterial (*DH5α E.coli*) transformation. The resulting colonies were grown overnight in liquid media and used as templates for PCR using vector primers. One microliter of the resulting PCR product was used for DNA sequencing using standard protocols of the ThermoSequenase II dye terminator cycle sequencing kit (Amershan-Pharmacia Biotech) and the reactions run on a MegaBACE 1000 automated sequencer.

### Computational analysis

A set of 10,092 ORESTES from different developmental stages of *Drosophila melanogaster *were generated. These sequences were submitted to an automated protocol for checking sequence quality, trimming to exclude vector and primer sequences, removing mtDNA, rRNA, bacterial and yeast and masking repetitive elements resulting in a total of 9,081 ORESTES (GeneBank_Accn EG974084 to EG974151 and ES688489 to ES697501): 360 from embryos at various stages (DE), 2,207 from larvae plus prepupae and pupae (DL), 4,490 from adults (DA) and 2,024 derived from a RNA mixture from embryos, larvae, pre-pupae, pupa and adults (DP). To assess the quality values of the sequences we used phred [36,37]. "N" nucleotides were trimmed by using a PERL script (cleanN). Sequences corresponding to pUC18 and primers were identified by crossmatch (cleanup_vector). rRNA and mtDNA sequences were identified using a program based on FASTA3 [38] and bacterial and yeast DNA sequences were identified by BLAST [39]. The databases for rRNA, mtDNA, bacterial DNA (*E.coli*) and yeast were compiled from GenBank. These ORESTES were processed with the assembly tool Cap3 [40]. Clustering of the 9,081 ORESTES resulted in 1,303 non-redundant clusters: 575 contigs plus 728 singletons. The 1,303 obtained clusters were submitted to automated BLASTn search against the release 1 annotated genes [1] resulting in the identification of 176 unannotated ORESTES clusters. Clustering of the ORESTES generated from each library resulted in 113 non-redundant clusters from DE, 430 from DL, 887 from DA and 729 from DP libraries.

### RNA extraction and northern blot analysis

*Drosophila *total RNA was extracted from embryo, larvae plus prepupae and pupae or adults. After homogenization in lysis buffer (10 mM Tris-HCl, 2% SDS, 50 mM EDTA, 5% ethanol, pH 9.0), total RNA was extracted by adding 10 vol of Trizol, following the manufacter's instructions (Invitrogen). RNA PolyA^+ ^was obtained using the Oligotex kit (Qiagen). The RNA was fractionated in 1% agarose formaldehyde-denaturing agarose gels and blotted to nylon membranes (Hybond N, Amershan, UK). RT-PCR, cloning, northern blotting, probe labelling, hybridization and post hybridization washes were performed essentially as described in Sambrook et al. [41]. The final washes were performed at 65°C in the presence of 0.1× SSC and 0.2% SDS. Primers used for SP212 cloning: Foward-exon1-1: 5' TCA GTC TTA TTT GCC CAC CG 3'; Foward-exon1-2: 5' GTG TCG CTA ATC GCC TTG G 3'; Foward-exon2-1: 5' GCC TCC GCC GTG GGT TCC 3'; Reverse-exon2-1: 5' CCG ATC CTG TAA GCT GTC G 3'; Reverse-exon3-ORESTES: 5' TGC CAC GGG ATG AGG TAG G 3'; Reverse-exon4-1: 5' ACA GGG TCC AGA TCC ATC G 3'; Reverse-exon4-2: 5' CTG ATT AAG CTG GCA GGT GC 3'.

### Infecting experiments

The insects were challenged by pricking with sharpened needles, which had been previously dipped into concentrated cultures of microorganisms. In the asceptic pricking the needles were first disinfected by ethanol. **Microorganisms**. *E.coli *(Gram^-^) was cultured in LB medium and *Staphylococcus aureus***(**Gram^+^) was grown in BHI medium (a gift from P.S.R. Coelho). *Aspergillus fumigatus *was grown on Sabouraud-agar medium. Spores and hyphae were harvested in saline (a gift from Dr. C. Maffei).

## Authors' contributions

RMM participated in ORESTES cloning and sequencing, carried out part of the *in silico *analysis and performed the molecular experiments, VV participated in ORESTES generation and *in silico *analysis and helped to prepare the manuscript, EDN, EME, NM, RGRP participated in generation of ORESTES profiles, JFS, DDA helped to perform the ORESTES cloning and sequencing, MAVC, WASJr and SJS developed bioinformatic tools for the ORESTES databank analysis, AJGS and MAZ conceived the study, MLPL participated in the design and coordination of the study and prepared the manuscript. All authors read and approved the final manuscript.
